# Trends in incidence and average waiting time for arthroplasty from 1998–2021: an observational study of 282,367 patients from the Scottish arthroplasty project

**DOI:** 10.1308/rcsann.2023.0039

**Published:** 2023-06-27

**Authors:** M Jabbal, J Burt, J Clarke, M Moran, P Walmsley, PJ Jenkins

**Affiliations:** ^1^ Royal Infirmary of Edinburgh, UK; ^2^ Golden Jubilee National Hospital, UK; ^3^ Victoria Hospital Kirkcaldy, UK; ^4^ Glasgow Royal Infirmary, UK

**Keywords:** Hip replacement arthroplasty, Knee replacement arthroplasty, Waiting list, Waiting lists

## Abstract

**Background:**

Current waiting times for arthroplasty are reported as being the worst on record. This is a combination of increasing demand, the COVID-19 pandemic and longer standing shortage of capacity. The Scottish Arthroplasty Project (SAP) is a National Audit that analyses all joint replacements undertaken in the Scottish NHS and Independent Sector. The aim of this study was to investigate the long-term trend in provision and waiting time for lower limb joint replacement surgery.

**Methods:**

All total hip replacements (THR) and total knee replacements (TKR) undertaken in NHS Scotland from 1998 to 2021 were identified. Waiting times data were analysed each year to determine the minimum, maximum, median, mean and standard deviation.

**Results:**

In 1998, there were 4,224 THR and 2,898 TKR with mean (range, SD) waiting time of 159.5 days (1–1,685, 119.8) and 182.9 days (1–1,946, 130.1). The minimum waiting times were both in 2013 for 7,612 THR – 78.8 days (0–539, 46) and 7,146 TKR – 79.1 days (0–489, 43.7). The maximum waiting times recorded were in 2021 with 4,070 THR waiting 283.7 days (0–945, 215) and 3,153 TKR waiting 316.8 days (4–1,064, 217).

**Conclusions:**

This is the first robust large-scale national dataset showing trends in incidence and waiting time for THR and TKR over two decades. There was an expansion of activity with a reduction in waiting time, which peaked in 2013, followed by an increase in waiting time with a plateau and modest decline in the number of procedures.

## Introduction

In the 1990s, the first targets for treatment were implemented in the UK, guaranteeing a maximum two-year wait for non-emergency surgery such as arthroplasty, and reducing rates of death from specific diseases.^1^ This was reinforced in the 2000s with increased investment in healthcare and performance review, often publicising underperforming healthcare boards.^2^ Significant improvements were seen, with subsequent targets stating patients should not wait longer than 12 weeks for planned treatment.^3^ In the following years, improvements were under threat from acute services requiring more resources such as inpatient beds, which lead to annual cancellations during winter.^4^

Waiting times after 2010 were seen to plateau or increase in the UK and other countries in the Organisation for Economic Cooperation and Development (OECD).^5^ During this time period, demand for arthroplasty has been rising steadily and is expected to continue. Analysis of the National Joint Registry of England and Wales has modelled an increase of 117% and 134% for total knee arthroplasty (TKA) and total hip arthroplasty (THA), respectively, by 2030. Factors such as an ageing population, longer time in employment and higher expectation of having arthritis treated to maintain lifestyle contribute to this increasing demand.^6^

Disruption of elective orthopaedics as a result of the COVID-19 pandemic has lead to waiting lists for hip and knee replacement being the largest on record, with over 730,000 patients in England and Wales awaiting surgery.^7^ Whereas all surgical specialties face similar issues, it was observed that orthopaedics had more cancellations over the period, with up to 82% of all cases being cancelled.^8^ Health boards across the UK have recently released ambitious plans to reduce waiting times for patients awaiting elective surgery such as hip and knee replacement.^9,10^ These include abolishing two-year waits, 18-month waits and one-year waits over the coming years.^11^

There are many accounts in patient support websites, such as Versus Arthritis,^12^ and in the media in general about increasing waiting times^13^; however, there are no published reports examining similar trends over the last 10 years.

The primary aim of this study was to examine the trends in average waiting time for hip and knee replacement over the time period of 1998–2021. A secondary aim was to examine the volume of arthroplasty over time.

## Methods

This study used data held in the Scottish Arthroplasty Project (SAP) dataset, a national audit that monitors the outcome of all arthroplasty procedures undertaken in all NHS Scotland. Data from independent-sector hospitals also began to be collected voluntarily at a later date. The SAP process of continuous audit, quality improvement and identification of outliers has been previously described.^14^

SAP receives data from two routinely collected administrative datasets: the Scottish Morbidity Record (SMR) 01 and the National Records of Scotland NHS Central Register of Deaths. SMR01 is an episode-based patient record relating to all inpatients and day cases discharged from non-obstetric and non-psychiatric specialties. It is similar to the HES dataset in NHS England and Wales. Data are collected by local coding teams and submitted to the Information Services Division (ISD) of NHS Public Health Scotland. A record is generated when a patient completes an episode of inpatient or daycase care. Examples include discharge home from hospital, transfer to another clinician (either at the same or a different hospital), a change of specialty (either under the same or a different clinician) or death. There are now over 1,000,000 SMR01s generated each year. Data are audited every 42 days (6 weeks) from patient discharge, checking for completeness.^15^

Data collected include patient identifiable and demographic details, episode management details and general clinical information. Currently diagnoses are recorded using the ICD-10 classification and operations are recorded using the OPCS-4 classification. Information such as waiting time for inpatient per daycase admission and length of stay may be derived from the episode management data. SMR01 data linkage is performed via a unique identifier given to every person living in Scotland – the community health index (CHI).

A search of the entire SAP dataset was performed. Inclusion criteria were all primary THA and TKA performed in the time period of 1998–2021. Exclusion criteria were any procedures before 1998 and revision procedures. Before 1998, waiting times were available; however, they were not routinely quality checked. All data from 1998 onwards were audited and checked in the manner described above. The minimum completeness was stated as >90% by the information analysts. Approval to undertake this study was obtained from the SAP Steering Committee and NHS Public Health Scotland. Under Health Research Authority guidance, no secondary ethical approval was required.

Data were analysed and presented with Microsoft Excel.

## Results

### Incidence per 100,000 population

**Figure 1:**
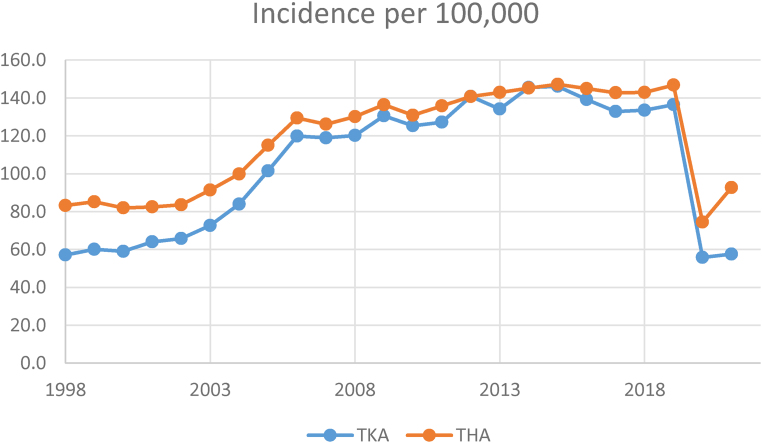
Incidence per 100,000 of population. THA = total hip arthroplasty; TKA = total knee arthroplasty

In 1998, the Scottish population was 5,077,070, and the incidence per 100,000 of TKA and THA were 57.1 and 83.2, respectively. For TKA, this increased steadily to a maximum incidence of 146.1 in 2015, after which it declined, with a minimum incidence of 42.7 in 2021. For THA, the incidence grew steadily from 1998 to a maximum of 147.2 in 2015; this then plateaued until 2020. The minimum incidence was in 2021, with 35.3 procedures performed per 100,000 population (Table 1, Figure 1).

**Table 1:** Scotland population and incidence per 100,000

Year	Population	TKA	THA
1998	5,077,070	57.1	83.2
1999	5,071,950	60.1	85.2
2000	5,062,940	59.0	81.9
2001	5,064,200	64.0	82.5
2002	5,066,000	65.8	83.6
2003	5,068,500	72.6	91.4
2004	5,084,300	83.9	99.8
2005	5,110,200	101.5	115.0
2006	5,133,000	119.8	129.4
2007	5,170,000	118.9	126.1
2008	5,202,900	120.2	130.1
2009	5,231,900	130.5	136.4
2010	5,262,200	125.3	130.8
2011	5,299,900	127.2	135.8
2012	5,313,600	140.8	140.8
2013	5,327,700	134.1	142.9
2014	5,347,600	145.5	145.2
2015	5,373,000	146.1	147.2
2016	5,404,700	139.1	144.9
2017	5,424,800	132.8	142.7
2018	5,438,100	133.5	142.9
2019	5,463,300	136.4	146.8
2020	5,466,000	54.2	44
2021	5,479,900	42.7	35.4

THA = total hip arthroplasty; TKA = total knee arthroplasty

### Total hip arthroplasty

In 1998, 4,224 procedures were performed. This rose steadily to maximum activity in 2019, with 8,018 procedures performed. The minimum activity was in 2020, with 4,070 procedures performed.

**Figure 2:**
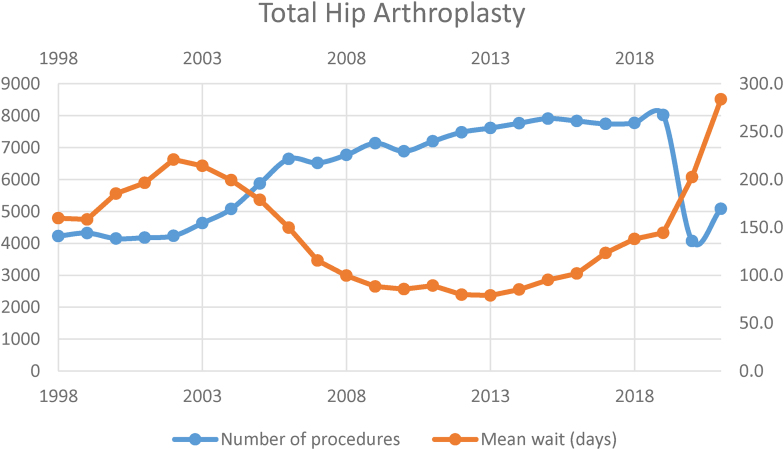
Total hip arthroplasty volume and waiting times1998–2021

In 1998, the mean waiting time was 159.5 days, which increased steadily to 220.8 days in 2002. After this, waiting time fell steadily until a minimum of 78.8 days in 2013—a 56% reduction since records began. The waiting time then increased gradually, with the last pre-covid era mean of 144.3 days in 2019. This represented an 83.1% increase in the time period 2013–2019. In 2021, the mean wait was 283.7 days, which represented a rise of 96.6% in the time period 2019–2021 (Table 2, Figure 2).

**Table 2:** Total hip arthroplasty volume and waiting times 1998–2021

Year	Number of procedures	Minimum wait (days)	Maximum wait	Mean wait	Median wait	Standard deviation
1998	4,224	1	1,685	159.5	133	119.8
1999	4,320	0	949	158.3	138	113.1
2000	4,148	0	1,127	185.3	167	124.4
2001	4,176	1	1,659	196.4	175	139.1
2002	4,234	0	2,010	220.8	198	155.1
2003	4,632	0	2,685	214.2	196	160.4
2004	5,076	1	1,700	199.3	188	137.9
2005	5,876	1	1,790	178.7	166	134.6
2006	6,642	1	1,376	149.7	135	104.9
2007	6,519	1	1,735	115.3	100	95.6
2008	6,769	0	1,203	99.6	95	59.7
2009	7,136	0	578	88.2	80	48.5
2010	6,884	1	627	85.4	72	51.7
2011	7,196	0	1,669	89.1	75	62.9
2012	7,479	0	683	79.7	68	50.2
2013	7,612	0	539	78.8	70	46.0
2014	7,763	0	431	85.1	78	44.7
2015	7,908	0	851	95.1	83	50.7
2016	7,833	0	582	101.7	88	54.0
2017	7,742	0	601	123.2	107	69.3
2018	7,772	0	579	137.8	119	78.8
2019	8,018	0	683	144.3	122	86.0
2020	4,070	0	768	202.5	189	122.7
2021	5,079	0	945	283.7	213	214.0

### Total knee arthroplasty

In 1998, 2,898 procedures were performed. This rose steadily to maximum activity in 2015, with 7,850 procedures performed, after which numbers declined slightly. The minimum activity was in 2020, with 3,049 procedures performed.

**Figure 3:**
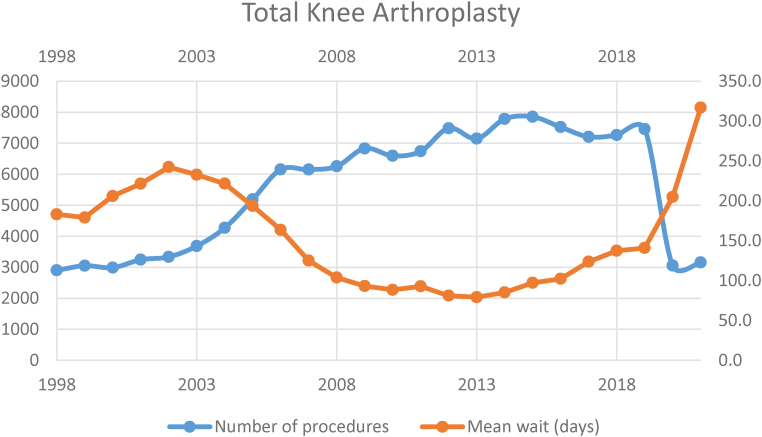
Total knee arthroplasty volume and waiting times 1998–2021

In 1998, the mean waiting time was 182.9 days, which increased steadily to 242.2 days in 2002. After this, waiting time fell steadily until a minimum of 79.1 days in 2013. This began to rise again with the last pre-covid era mean of 140.7 in 2019 (78% increase in period 2013–2019). The maximum wait of 316.8 days was in 2021 (125% increase from 2019–2021) (Table 3, Figure 3).

**Table 3:** Total knee arthroplasty volume and waiting times 1998–2021

Year	Number of procedures	Minimum wait (days)	Maximum wait	Mean wait	Median wait	Standard deviation
1998	2,898	1	1,946	182.9	158	130.1
1999	3,047	0	1,008	179.0	161	122.5
2000	2,987	0	1,445	205.9	187.5	130.1
2001	3,242	0	1,521	221.3	208	144.2
2002	3,332	1	1,563	242.2	231	151.2
2003	3,682	0	1,477	232.7	216	158.5
2004	4,268	1	2,009	221.5	213	144.5
2005	5,187	0	1,487	193.5	176	140.7
2006	6,151	0	1,947	163.4	143	123.8
2007	6,148	0	1,413	124.9	105	105.3
2008	6,253	0	1,210	103.7	97	61.2
2009	6,830	0	700	93.0	82	53.1
2010	6,594	0	1,112	88.3	74	55.5
2011	6,740	0	1,153	92.6	79	57.2
2012	7,481	0	619	80.9	68	52.6
2013	7,146	0	489	79.1	71	43.7
2014	7,781	0	528	85.0	78	44.7
2015	7,850	0	531	97.0	84	48.7
2016	7,520	0	520	102.1	87	54.5
2017	7,205	0	678	123.5	109	70.1
2018	7,261	0	645	137.3	122	77.0
2019	7,454	0	604	140.7	120	83.6
2020	3,049	2	747	204.8	195	121.9
2021	3,153	4	1,064	316.8	258	217.0

## Discussion

The current study has demonstrated a gradual increase in THA volume, which peaked in 2019 followed by a sharp reduction in 2020. Average waiting time decreased gradually from 1998 until 2013, then increased, with a sharp increase seen in 2020. For TKA, the volume increased from 1998 until 2015, after which it decreased, with a sharp reduction in 2020. Average waiting time decreased from 1998 until 2013, after which it increased.

Cooper *et al* demonstrated a similar trend in England in patients from 1997 to 2007, reporting that both THA and TKA waiting times reduced significantly from 260 to 140 days for TKA and from 210 to 125 days for THA.^16^ The authors describe that the government current at the time had invested heavily in healthcare, introducing waiting time targets and performance reviews dubbed “targets and terror”.^2^ To the best of the authors’ knowledge no published literature exists for comparison demonstrating trends in waiting list time for the period after 2007.

A steady increase in the annual volume of TKA and THA has been reported in this study, with incidence rising from 51.7 and 83.2 per 100,000 (1998) to 146.1 and 147.2, respectively (2015). In 2002, the National Waiting Times Centre opened in Scotland and provided extra theatre and consultant capacity.^17^ It allowed all health boards in the country to send patients with extended waiting times for surgery and is currently performing the highest volume of arthroplasty of all Scottish hospitals. Assessment of each individual health board capacity over the course of the study period is beyond the scope of this study. No accurate record exists of private sector activity trends during this time period. Yapp *et al* forecasted for the same population that demand would increase by 11.4% for TKA and 10.7% for THA by 2030.^18^ Similar levels have been reported by Wells *et al.* In Australia, primary THA increased from 50.9 (1994) to 60.9 (1998) per 100,000; TKA increased from 56.4 to 76.8 per 100,000.^19^ In the Swedish Knee arthroplasty register, Nemes *et al* reported that, in 1975, only 128 primary knee arthroplasties were performed (3.5 per 100,000 of the population); by 2013, 13,338 operations were performed (149.7 operations per 100,000).^20^ The incidence of THA was 25 in 1970, increasing to 332 per in 2010. They project that by 2030 these figures will be 210.1 (TKA) and 189.1 (THA).^21^ Jimenez *et al* report 161,791 patients having undergone THA from 2001 to 2008 in Spain. Incidence increased from 99 to 105 THA per 100,000 inhabitants from 2001 to 2008.^22^ Pilz *et al* report a much higher incidence of THA in Germany over the years 2010–2016, with 261 per 100,000 in 2010 increasing to 283 in 2016 and estimating this will rise by 27% by 2040.^23^ The findings of the current study are in keeping with these international reports; however, the further dimension of waiting time is added. It is currently difficult to effectively model how waiting times will develop as demand continues to increase; however, many health boards are well below pre-covid levels of productivity.^24^ A further concern is that extreme pressures in acute services are overwhelming many centres where elective care is also performed, leading to cancellations and increased waiting times. This phenomenon existed pre-covid and was referred to as “winter pressures”; however, these are now occurring much earlier in the year than expected.^25^ This unmet increasing demand risks waiting times spiralling out of control.

Waiting times are viewed as a key determinant of satisfaction with public services and variations geographically contribute to health inequality.^26,27^ Increases in waiting times are frequently in the news and media,^28^ with patient advocate groups such as “Versus Arthritis” releasing position statements on maximum waiting times.^12^ Endstage osteoarthritis is a severely debilitating disease, with increased time waiting for joint replacement being associated with increased pain level, deteriorating function and a considerable loss of quality-adjusted life years.^29^ Scott *et al* demonstrated there were patients waiting for total joint arthroplasty who had an EQ-5D general health questionnaire score so low their quality of life was considered “worse than death” (WTD).^30^ They also noted a rise in mean waiting time over the study period (2014–2017). Although this did not predict status, the increase in waiting times over the study period was mirrored by a rise in the proportion of patients defined as WTD. Clement *et al* reported during covid elective orthopaedic cancellations, the number of patients WTD had almost doubled, with increasing length of time on the waiting list associated with decreasing quality of life.^31^ Farrow *et al* have shown that opioid use may be increasing in those awaiting joint replacement as a result of longer waiting times,^32^ with negative effects such as increased risk of surgical complications and lower patient-reported outcome scores postoperatively.^33,34^ Given this growing body of literature on the negative associations with increased waiting times, the current study demonstrating a national trend prepandemic of increased waiting time is a cause for concern.

A strength of this study is the utilisation of a large, prospectively collected and quality-checked National dataset. This has allowed a long study period over two decades, and a large number of patients. The limitations of this study are lack of demographical details for the patients, and lack of analysis of association between waiting time and clinical profiles. These are potential areas for future work.

## Conclusion

This is the first analysis from a large-scale national dataset showing trends in waiting time for arthroplasty in the Scottish NHS over a 23-year period. Following a period of significant expansion of capacity, there was a reduction in waiting times that reached a trough in 2013. With increasing demand outstripping capacity, followed by the effects of the global COVID-19 pandemic, there has been a catastrophic decline in activity and consequent rise in waiting times to record levels.
